# Pigmentación exógena por nitrato de plata: aspectos dermatológicos y toxicológicos, a propósito de un caso

**DOI:** 10.7705/biomedica.5876

**Published:** 2021-06-15

**Authors:** Ángela Londoño, Camila Pérez, Rodrigo Restrepo, Nathalie Morales, Miguel Martínez, Daniela Morales

**Affiliations:** 1 Grupo de Investigación, Universidad CES, Medellín, Colombia Universidad CES Universidad CES Medellín Colombia

**Keywords:** nitrato de plata, argiria, melanoma, informes de casos, Colombia, Silver nitrate, argyria, melanoma, case reports, Colombia.

## Abstract

La pigmentación exógena por nitrato de plata es una enfermedad poco frecuente, cuyas manifestaciones clínicas pueden aparecer años después del contacto, lo que en ocasiones dificulta su diagnóstico. Se caracteriza por la presencia de máculas o placas azul-grisáceas en la piel o las mucosas de la zona de contacto que, en ocasiones, son muy similares a las lesiones melanocíticas y al melanoma, sus principales diagnósticos diferenciales.

Se reporta el caso de un paciente de Medellín, Colombia, con antecedentes familiares de melanoma y presencia de estas lesiones en todo el cuerpo.

## Abstract (en)

Exogenous pigmentation by silver nitrate is a rare disease whose clinical manifestations appear even years after the contact, making its diagnosis difficult on occasions. It is characterized by the presence of blue-gray macules or plaques on the skin or mucosa in the contact area, sometimes very similar to melanocytic lesions and melanoma, which constitute the main differential diagnosis.

We report the case of a male patient from Medellín, Colombia, with a family history of melanoma and the presence of these lesions throughout his body.

La pigmentación exógena por nitrato de plata es una enfermedad muy rara causada por el depósito de partículas de plata, con compromiso únicamente del estrato córneo de la epidermis. Las manifestaciones clínicas se pueden presentar incluso años después de la exposición a la plata de forma directa, ocupacional, por inhalación o ingestión (1). Pocos casos se han reportado en la literatura mundial, todos extrapolados de los de argiria cutánea localizada o generalizada.

## Caso clínico

Se presenta el caso de un paciente de 25 años con antecedentes familiares en primer y segundo grado de consanguinidad afectados por melanoma, múltiples carcinomas basocelulares, escamocelulares y nevus displásicos. Consultó por presentar lesiones maculares de una semana de evolución, asociadas con sensación urente y prurito, que aparecieron inicialmente en el borde radial de la muñeca derecha y se extendieron al tórax, el abdomen, los brazos, las piernas, las palmas y las plantas, un día después del inicio del cuadro clínico. El paciente negó otros síntomas.

**Figura 1:**
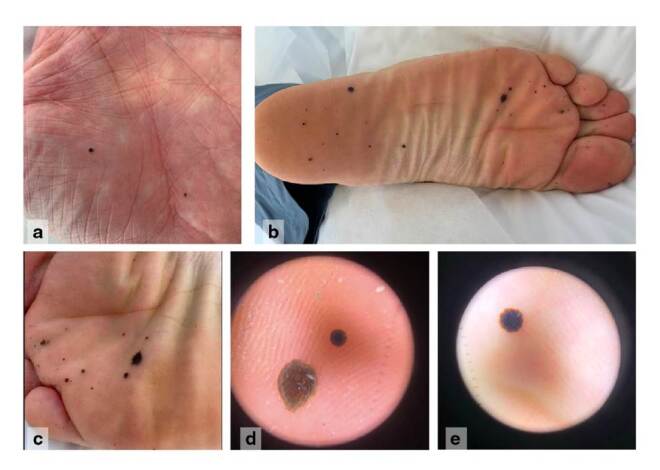
(a) Maculas cafés, múltiples y bien definidas en la palma de la mano izquierda. (b-c) Máculas cafés, múltiples y bien definidas en la planta izquierda. (d-e) Mácula hiperpigmentada, bien definida, sin red de pigmento en la dermatoscopia

**Figura 2:**
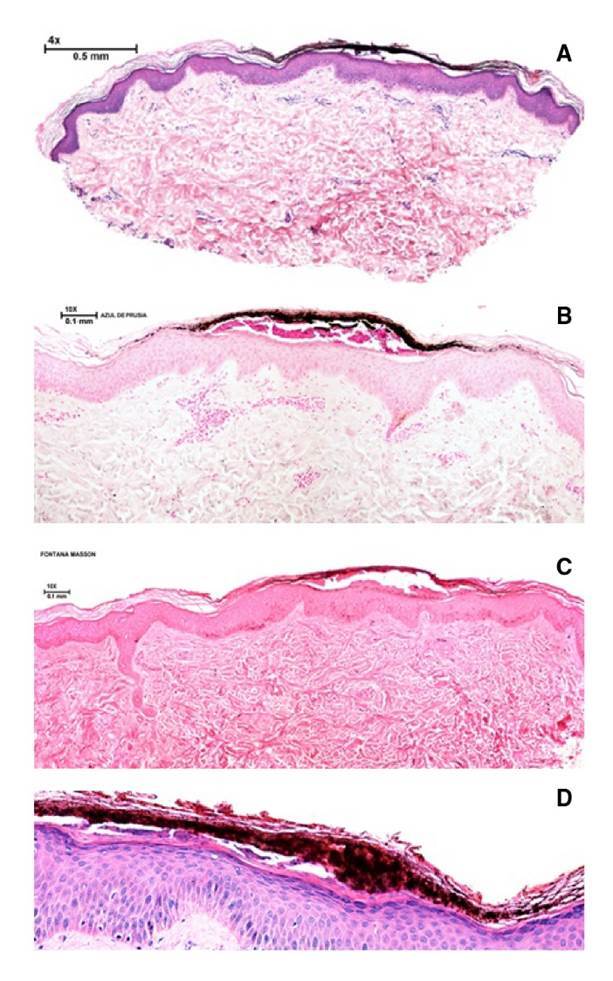
Histopatología. **(a)** Se observa condensación de queratina y depósito de pigmento pardo- negruzco en la capa córnea, que no afecta en mayor medida la epidermis ni la dermis; únicamente hay un discreto infiltrado inflamatorio linfocitario perivascular superficial. Hematoxilina y eosina, 4X. **(b)** Se observa el pigmento en la capa córnea, asociado con una discreta paraqueratosis. Hematoxilina y eosina, 2X. **(c)** Coloración negativa para hemosiderina en el pigmento estudiado. Azul de Prusia, 20X. **(d)** Coloración positiva para melanina en forma de gránulos negruzcos en el pigmento estudiado. Tinción de Fontana-Masson, 20X.

En el examen físico presentaba múltiples lesiones maculares, hiperpigmentadas, bien definidas, de forma variable y tamaño de 0,3 a 0,6 mm, y sin red de pigmento en la dermatoscopia (figura 1). Se sospechó un cuadro clínico de pigmentación exógena *Vs*. melanoma y se tomó una biopsia de una de las lesiones. En la histopatología se observó la presencia de glóbulos negro-cafés en la capa córnea de la epidermis, con discreto infiltrado linfocitario y paraqueratosis (figura 2). La tinción de Fontana-Masson fue positiva, en tanto que la de azul de Prusia y la detección del gen *SOX-10* fueron negativas.

Los hallazgos clínicos e histopatológicos son indicativos de pigmentación exógena por nitrato de plata. Al interrogar nuevamente al paciente, este refirió una posible exposición al metal el día anterior a la aparición de las lesiones, cuando un recipiente con nitrato de plata se quebró accidentalmente y él pudo haber tenido un contacto inadvertido con el metal, lo que explicaría las lesiones. Se le practicaron diversos exámenes de laboratorio, con los cuales se descartó compromiso multisistémico.

Se le presentaron varias opciones de tratamiento tópico que el paciente no siguió. Posteriormente, se produjo el proceso descamativo y las máculas desaparecieron espontáneamente tres semanas después de iniciado el cuadro clínico.

## Discusión

El uso de la plata como medicamento data del año 980 a. C., cuando Avicena la describió como un purificador sanguíneo útil para contrarrestar la dificultad respiratoria y las palpitaciones. En la descripción de patología de la muerte de Avicena, mencionan una “pigmentación azulada” de sus ojos, siendo este, probablemente, el primer caso mencionado de argiria (2). En los siglos XIX y XX, la plata se empezó a comercializar como pócima curativa para todo tipo de enfermedades: epilepsia, gastroenteritis, infecciones, HIV y cáncer. En 1999, la *Federal Drug Administration* (FDA) de Estados Unidos anunció que los productos que contenían plata no se consideraban seguros ni efectivos; sin embargo, siguen apareciendo casos debido a la exposición ocupacional y a su uso en la medicina alternativa (3).

En la medicina moderna, el primer caso reportado e indexado de argiria cutánea localizada data de 1952 y, a partir de este, se han reportado 74 casos en total, entre los que sobresalen diez relacionados con la acupuntura, diez con la aplicación o ingestión del metal, siete asociados con pseudoocronosis, nueve casos de lesiones confundidas con nevus azul, tres asociados con antecedentes personales de melanoma, uno de ellos con lesiones metastásicas en la piel y otro en el que había antecedentes familiares de melanoma. El reporte de casos de pigmentación exógena por nitrato de plata es, incluso, más escaso (4).

En el presente caso se trata de un paciente con pigmentación exógena por nitrato de plata en quien llamaba la atención la pigmentación oscura bien definida, el corto tiempo de evolución después del contacto y la desaparición espontánea de las lesiones, lo cual implicaba un contacto directo con el metal. Debido a las condiciones del paciente, se le preguntó sobre exposiciones que explicaran los hallazgos patológicos y descartaran la posibilidad de melanoma como diagnóstico diferencial; se descubrió que había estado expuesto al nitrato de plata y el contacto había pasado desapercibido. Características como la ausencia de red de pigmento en la dermatoscopia, la rápida evolución de las lesiones y el compromiso únicamente a nivel de la capa córnea de la epidermis, favorecieron el diagnóstico.

Las lesiones de pigmentación exógena por nitrato de plata se han descrito incluso años después del contacto por inhalación, ingestión o exposición ocupacional o directa (5). El contacto con la plata puede darse en actividades como joyería, fotografía, histotecnología, manejo de baterías o pintura, floristería, cosmética, industria electrónica y automotriz, soldadura, uso de sulfadiazina (4), apósitos y prótesis recubiertas (6), amalgamas, agentes hemostásicos (7), en el tratamiento del tabaquismo (8), la acupuntura (9) y el uso de plata coloidal (10,11).

La absorción de la plata depende del tipo de compuesto, el periodo de exposición y los cambios en el sitio de entrada (2). En la piel intacta no se ha logrado demostrar la absorción sistémica, pero cuando hay alteración en la barrera de la piel, podría generarse una pigmentación superficial con plata fotorreducida que forma complejos proteínicos muy insolubles, los cuales permanecen más tiempo en el tejido (10,12,13). La excreción depende de la presentación: cuando es cutánea, la excreción ocurre por recambio epidérmico (14) y, si es sistémica, se elimina en la orina y en las heces (2,12).

Cuando el contacto es directamente con la piel, la plata ingresa por los conductos de las glándulas sudoríparas y se deposita en la porción secretora cerca de la unión dermo-epidérmica, en forma de sulfuro o selenio de plata, el cual es capturado por las fibras elásticas y desencadena la oxidación de componentes solubles en los tejidos, lo cual impide la inhibición de la tirosinasa y aumenta la producción de melanina, con la consecuente pigmentación característica de la argiria cutánea localizada (15,16).

Sin embargo, en la pigmentación exógena por el metal, se compromete únicamente la capa córnea por la formación de cloruro de plata con el cloro del sudor, el cual es reducido por la luz ultravioleta para formar partículas coloides de plata metálica, sin comprometer el melanocito (17).

La manifestación en la piel es una pigmentación azul-grisácea, negra o café, en forma de máculas o placas, bien o mal definidas, que pueden afectar también las mucosas según la zona de contacto (9).

El cuadro clínico permite diagnosticar los diferentes tipos de argiria cutánea (localizada o generalizada) y la pigmentación exógena por nitrato de plata. El diagnóstico se confirma por histopatología y, en algunas ocasiones, por microscopía electrónica. En la argiria cutánea localizada, la histopatología muestra glóbulos negro-cafés en la dermis adheridos a las fibras elásticas, las paredes de los vasos sanguíneos y los nervios, las glándulas sudoríparas, las sebáceas y el tejido conjuntivo de los folículos pilosos (7,18); se diferencia de la pigmentación exógena, la cual es un proceso mucho más localizado que compromete solamente la epidermis, más específicamente, el estrato córneo, sin alterar otras estructuras (17).

La microscopía de campo oscuro permite visualizar un patrón en “cielo estrellado” en el que los glóbulos del metal adquieren un aspecto brillante. Mediante la microscopía electrónica se demuestra la presencia de la plata y su composición química se determina con espectroscopia por dispersión de energía para diferenciarla de otros metales (17).

El diagnóstico diferencial de la pigmentación exógena por nitrato de plata incluye la argiria cutánea, especialmente en su forma localizada, los nevus azules, los melanocíticos, los tatuajes, el melanoma y la intoxicación por otros metales pesados, como mercurio, bismuto, oro, arsénico y plomo (1,8).

En lesiones muy extensas, se debe descartar la argiria cutánea generalizada, la cual se caracteriza por el riesgo de toxicidad aguda, con un amplio rango de manifestaciones clínicas que van desde síntomas leves e inespecíficos, como vómito y diarrea, hasta falla multisistémica, neurotoxicidad, choque y muerte (19). Sin embargo, la condición no deja de ser rara y hay casos excepcionales de toxicidad debida al consumo típico del metal (0,007-0,5 µg/kg), por debajo de los niveles tóxicos reportados en animales (0,5 mg/kg)(12).

Además de la histopatología, existen otras ayudas diagnósticas que permiten hacer el diagnóstico. Con la microscopía confocal de reflectancia es posible diferenciar si se trata de melanoma, nevus melanocíticos o nevus azules. La ausencia de un patrón de anillos o de malla en la unión dérmico-epidérmica permite excluir el diagnóstico de nevo melanocítico, la ausencia de células pagetoides o células basales o dérmicas atípicas permite excluir el diagnóstico de melanoma, y la presencia de células dendríticas hiperrefráctiles dispuestas en nidos o en hojas y rodeadas por fibras de colágeno brillantes permite excluir el diagnóstico de nevus azul. Por otra parte, los tatuajes se caracterizan por la presencia de pigmentos de diferentes densidades, tamaños y distribución en el tejido (20).

También, se pueden utilizar paneles de marcadores inmunohistoquímicos como las proteínas S100, melan A y HMB-45, para descartar el diagnóstico de melanoma y diferenciarlo de condiciones como la argiria cutánea localizada y la pigmentación exógena. Estos marcadores se usan en conjunto debido a su falta de especificidad y sensibilidad cuando se usan por separado. El marcador melanocítico SOX-10, usado en el paciente reportado, es un factor de crecimiento de la cresta neural necesario para la especificación de los melanocitos y las células de Schwann, con excelente sensibilidad para el diagnóstico del melanoma cutáneo (21).

El tratamiento debe iniciarse con la eliminación del factor desencadenante (22). Por lo general, las lesiones desaparecen debido al recambio epidérmico al cabo de algunas semanas de haber suspendido el contacto, como ocurrió en el paciente reportado (17). Sin embargo, algunas pigmentaciones pueden volverse permanentes, por lo que se han explorado varias alternativas terapéuticas para disminuir o hacer desaparecer la pigmentación. Contra la pigmentación cutánea se han ensayado tratamientos con selenio, azufre, quelantes (23), decolorantes como la hidroquinona, y procedimientos como la dermoabrasión (24).

## Conclusiones

La escasa frecuencia de la exposición a la plata, el amplio espectro de manifestaciones clínicas que causa y sus diversas formas de presentación, que van desde la pigmentación exógena y la argiria cutánea localizada o generalizada hasta la toxicidad aguda, y los múltiples diagnósticos diferenciales que se deben considerar, hacen que el proceso para su diagnóstico sea muy exigente para el médico tratante. Por requerirse dosis muy altas para alcanzar su toxicidad, por lo general, sus efectos solo tienen implicaciones estéticas, por lo cual es importante conocer las manifestaciones clínicas, su toxicocinética y toxicodinamia, para comprender las implicaciones del contacto con la plata en la salud de los pacientes.

## References

[B1] Salvaneschi MB, Jaled M, Olivares L, Candiz ME, Maronna E (2017). Argiria cutánea generalizada por aplicación prolongada de sulfadiazina de plata. Dermatología Argentina.

[B2] Hill WR, Pillsburg DM (1939). Argyria; The pharmacology of silver.

[B3] Jung I, Joo EJ, Suh BS, Ham CB, Han JM, Kim YG (2017). A case of generalized argyria presenting with muscle weakness. Ann Occup Environ Med.

[B4] Enei ML, Paschoal FM, Valdés R (2013). Argyria mimicking a blue nevis: Dermoscopy features. An Bras Dermatol.

[B5] Okan D, Rg Sibbald (2007). So what if you are blue? Oral colloidal silver and argyria are out: Safe dressings are in. Adv Skin Wound Care.

[B6] Drake PL, Hazelwood KJ (2005). Exposure-related health effects of silver and silver compounds: A review. Ann Occup Hyg.

[B7] Fisher NM, Marsh E, Lazova R (2003). Scar-localized argyria secondary to silver sulfadiazine cream. J Am Acad Dermatol.

[B8] Glehr M, Leithner A, Friesenbichler J, Goessler W, Avian A, Andreou D (2013). Argyria following the use of silver-coated megaprostheses: No association between the development of local argyria and elevated silver levels. Bone Jt J.

[B9] Marshall JP, Schneider RP (1977). Systemic argyria secondary to topical silver nitrate. Arch Dermatol.

[B10] Macintire D, Mclay AL, East BW, Williams ED, Boddy K (1978). Silver poisoning associated with an antismoking lozenge. Br Med J.

[B11] Park MY, Lee JS, Jin HJ, You HS, Kim GW, Ko HC (2018). Localized argyria: Troublesome side-effect of acupuncture. J Eur Acad Dermatol Venereol.

[B12] Hadrup N, Lam HR (2014). Oral toxicity of silver ions, silver nanoparticles and colloidal silver - A review. Regul Toxicol Pharmacol.

[B13] Hadrup N, Sharma AK, Loeschner K (2018). Toxicity of silver ions, metallic silver, and silver nanoparticle materials after in vivo dermal and mucosal surface exposure: A review. Regul Toxicol Pharmacol.

[B14] Dempsey EW, Wislocki GB (1955). The use of silver nitratre as a vital stain, and its distribution in several mammalian tissues as studied with the electron microscope. J Biophys Biochem Cytol.

[B15] Matuk Y, Ghosh M, McCulloch C (1981). Distribution of silver in the eyes and plasma proteins of the albino rat. Can J Ophthalmol.

[B16] Furchner JE, Richmond CR, Drake GA (1968). Comparative metabolism of radionuclides in mammals-IV. Retention of silver-110m in the mouse, rat, monkey, and dog. Health Phys.

[B17] Posada A (2014). Pigmentación oscura del estrato córneo, ¿cuál es su diagnóstico?. Revista de la Asociación Colombiana de Dermatología y Cirugía Dermatológica.

[B18] McClain CM, Kantrow SM, Abraham JL, Price J, Parker ER, Robbins JB (2013). Localized cutaneous argyria: Two case reports and clinicopathologic review. Am J Dermatopathol.

[B19] Mirsattari SM, Hammond RR, Sharpe MD, Leung FY, Young GB (2004). Myoclonic status epilepticus following repeated oral ingestion of colloidal silver. Neurology.

[B20] García-Martínez P, López-Aventín D, Segura S, Gómez-Martín I, Lloreta J, Ibáñez J (2016). In vivo reflectance confocal microscopy characterization of silver deposits in localized cutaneous argyria. Br J Dermatol.

[B21] Alghamdi SA, Zoroquiain P, Dias ABT, Alhumaid SR, Aldrees S, Burnier MN (2015). Diagnostic value of SOX-10 immunohistochemical staining for the detection of uveal melanoma. Ecancermedicalscience.

[B22] Griffiths MR, Milne JT, Porter WM (2006). Penile argyria. Br J Dermatol.

[B23] Liu J, Wang Z, Liu FD, Kane AB, Hurt RH (2012). Chemical transformations of nanosilver in biological environments. ACS Nano.

[B24] Tajirian AL, Campbell RM, Robinson-Bostom L (2006). Localized argyria after exposure to aerosolized solder. Cutis.

